# Retrieval of Cu^2+^ and Cd^2+^ ions from aqueous solutions using sustainable guar gum/PVA/montmorillonite nanocomposite films: effect of temperature and adsorption isotherms

**DOI:** 10.3389/fchem.2024.1393791

**Published:** 2024-08-05

**Authors:** Haifa A. S. Alhaithloul, Ibtisam Mohammed Alsudays, ElSayed G. Zaki, Shymaa M. Elsaeed, Amal E. Mubark, Lurana Salib, Gehan Safwat, Gniewko Niedbała, Ayman Diab, Mohamed A. Abdein, Afaf Alharthi, Shadi A. Zakai, Amr Elkelish

**Affiliations:** ^1^ Department of Biology, College of Science, Jouf University, Sakaka, Saudi Arabia; ^2^ Department of Biology, College of Science, Qassim University, Burydah, Saudi Arabia; ^3^ Egyptian Petroleum Research Institute, Cairo, Egypt; ^4^ Semi-Pilot Plant Department, Nuclear Materials Authority, Cairo, Egypt; ^5^ Faculty of Biotechnology, October University for Modern Sciences (MSA), 6th of October, Egypt; ^6^ Department of Biosystems Engineering, Faculty of Environmental and Mechanical Engineering, Poznan University of Life Sciences, Poznań, Poland; ^7^ Seeds Development Department, El-Nada Misr Scientific Research and Development Projects, Mansoura, Egypt; ^8^ Department of Clinical Laboratory Sciences, College of Applied Medical Sciences, Taif University, Taif, Saudi Arabia; ^9^ Department of Clinical Microbiology and Immunology, Faculty of Medicine, King Abdulaziz University, Jeddah, Saudi Arabia; ^10^ Department of Biology, College of Science, Imam Mohammad Ibn Saud Islamic University (IMSIU), Riyadh, Saudi Arabia; ^11^ Botany Department, Faculty of Science, Suez Canal University, Ismailia, Egypt

**Keywords:** wastewater, heavy metal, guar gum, polyvinyl alcohol, isotherms, nanocomposite

## Abstract

Uncontrolled or improperly managed wastewater is considered toxic and dangerous to plants, animals, and people, as well as negatively impacting the ecosystem. In this research, the use of we aimed to prepare polymer nanocomposites (guar gum/polyvinyl alcohol, and nano-montmorillonite clay) for eliminating heavy metals from water-based systems, especially Cu^2+^ and Cd^2+^ ions. The synthesis of nanocomposites was done by the green method with different ratios of guar gum to PVA (50/50), (60/40), and (80/20) wt%, in addition to glycerol that acts as a cross-linker. Fourier-transform infrared spectroscopy (FT-IR) analysis of the prepared (guar gum/PVA/MMT) polymeric nano-composites’ structure and morphology revealed the presence of both guar gum and PVA’s functional groups in the polymeric network matrix. Transmission electron microscopy (TEM) analysis was also performed, which verified the creation of a nanocomposite. Furthermore, theromgravimetric analysis (TGA) demonstrated the biocomposites’ excellent thermal properties. For those metal ions, the extreme uptake was found at pH 6.0 in each instance. The Equilibrium uptake capacities of the three prepared nanocomposites were achieved within 240 min. The maximal capacities were found to be 95, 89 and 84 mg/g for Cu^2+^, and for Cd^2+^ were found to be 100, 91, 87 mg/g for guar gum (80/20, 60/40 and 50/50), respectively. The pseudo-2^nd^-order model with R^2^ > 0.98 was demonstrated to be followed by the adsorption reaction, according to the presented results. In less than 4 hours, the adsorption equilibrium was reached. Furthermore, a 1% EDTA solution could be used to revitalize the metal-ion-loaded nanocomposites for several cycles. The most promising nanocomposite with efficiency above 90% for the removal of Cu^2+^ and Cd^2+^ ions from wastewater was found to have a guar (80/20) weight percentage, according to the results obtained.

## 1 Introduction

Climate change and global warming with overpopulation request non-conventional water resources very the incentive for wastewater reuse. But there are many challenges national and regional both where wastewater disposal to the environment is subject to regulated standards Wastewater reuse is encouraged by climate change, global warming, and overpopulation, which increase the demand for non-conventional water resources. But where wastewater disposal to the environment is subject to regulated norms, there are numerous obstacles on a national and regional level (Donat et al., 2016; Bretschger, 2020). Wastewater contains inorganic and metal ions pollutants and organic as pharmaceutical materials, drugs, and dyes (Kadiri et al., 2021; Bansalah et al., 2023; El Amri et al., 2023; Jebli et al., 2023; Lebkiri et al., 2023). The most dangerous of them are the heavy elements that negatively affect the environment, especially the soil and the marine environment all of this makes us move research toward the treatment of these contaminated elements Organic substances such as pharmaceutical components, medicines, and colors are found in wastewater together with inorganic and metal ions contaminants. The heavy elements that damage the ecosystem especially the soil and the marine environment are the most dangerous of them. We are concentrating our research on the treatment of these polluted materials as a result. These metals are a class of metals that include copper, zinc, chromium, nickel, and cadmium. Even in small amounts, these metals are toxic. Metal ions are due to many activities natural and industrial, such as soil corrosion and rock weathering. These metals, a category that includes cadmium, chromium, nickel, zinc, and copper, are hazardous even in small amounts. Numerous human and non-human activities, such as soil corrosion and rock weathering, produce metal ions (Wołowiec et al., 2019). (Natural factors, together with industrial ones are expected to be a part of the pollution processes and environmental metal ion toxicity. Natural factors include soil corrosion, soil weathering, and volcanic eruptions while the industrial factors include acid rain and metal piping Environmental metal ion toxicity and pollution processes are believed to involve both natural and industrial influences. In contrast to industrial influences like acid rain and metal pipelines, natural forces like soil corrosion, soil weathering, and volcanic eruptions.

The structure of guar gum is composed of two linked d-mannose units as the polymer’s keystone, with a d-galactose unit linked one to six to every second mannose unit. Throughout the guar gum polymer, the galactose-mannose unit is repeated. Guar gum has a 2:1 ratio of mannose to galactose unit. Guar gum easily hydrates at low concentrations in cold water to produce very viscous solutions; but, unless more polysaccharides are added, it does not gel (Zeece, 2020). It acts as a flocculant and a non-toxic alternative to the synthesis of polymers in the treatment of wastewater from the food industry. Polymer-based processes for treating oily water use three main mechanisms: flocculation, adsorption, and complexation (Lucas et al., 2009). The procedure for removal of dye may include precipitation and flotation (Liu et al., 2021; Han et al., 2022; Li et al., 2022). An alternate method for recovering dyes from aqueous solutions is sorption, which uses less expensive bio-based sorbents (Naushad et al., 2016; Bushra et al., 2021). GG films have the potential to be applied in a variety of adsorption fields because of their breaking biodegradability and permanent nature after their life cycle (Elgarahy et al., 2023).

A cross-linked network of guar gum having a permeable structure has grabbed the attention of heavy metals entrapment in aqueous solution (Sharma et al., 2013). Hydrolyzed guar gum had been manufactured which was oxidized using nitrogen oxides to their relevant polycarboxylic forms and was crosslinked using N, N-methylene-bis-acrylamide. The synthesized polymer hydrogels were strongly evaluated as an absorbent for Cu^2+^. The results demonstrated that hydrogels had an extraordinary absorption capacity of 125.893 mg per g, making them highly sorbent (Singh et al., 2009). Improved copolymer and grafted polymethyl acrylate onto guar gum were used to remove Cr (VI) from the wastewater industry. The copolymer product demonstrated remarkable efficiency, as evidenced by its 29.67 mg per g absorption capacity at pH 1.0. Polymethyl acrylate was grafted onto guar gum to remove Cr(VI) from the wastewater industry. The absorption capacity of the mixture was found to be 29.67 mg per g at pH 1.0. Furthermore, the scientific research on revitalization showed the ability to reuse of reusing the product for five cycles.

The unique aspect of this work is the use of green chemistry, which uses cleaner solvents and auxiliaries instead of organic solvents, to create guar films. The primary idea of green chemistry also depends on designing safer chemicals with eco-friendly components and employing less harmful chemical synthesizers when the synthesis is based on biopolymers like guar gum.

The use of green chemistry in the synthesis of guar films is what makes this work innovative where no organic solvent is used which provides safer solvents and auxiliaries. The main principle of green chemistry also relies upon using less hazardous chemical synthesizers where the synthesis is based on biopolymers such as guar gum, and designing safer chemicals where the materials are eco-friendly.

The present work aimed to synthesize nanocomposites (guar gum/polyvinyl alcohol, and nano-montmorillonite clay) using an inexpensive technique to test their effectiveness in the retrieval of Cd^2+^ and Cu^2+^ ions from liquid solutions in addition to investigating the used mechanism. Therefore, prepared nanocomposites were defined using various techniques. The effects of pH, temperature, time, and ion concentration were researched about adsorption. Conversely, the processes of regeneration and desorption were investigated in the loaded adsorbent. The kinetic data could be shaped with the use of the Lagergren equations. We used a variety of adsorption isotherm models to derive the equilibrium results.

## 2 Materials and methods

Guar gum (GG, Mw: 100 kDa), CAS#9000-30-0; The supplier of the polyvinyl alcohol (PVA) (Mwt ∼17–18 kDa) was an international chemical business while for glycerol (purity 99.5%, Mwt ∼92) was Al-Gomhoria Pharmaceutical Co. sodium montmorillonite (Na + -MMT) with a cation exchange mM/100 g (ACROS organics, Cairo, Egypt). Delhi, India is the source of cetyl trimethyl ammonium bromide (CTAB).

### 2.1 Methods

#### 2.1.1 Organo-montmorillonite (OMMT) preparation from MMT

A suspension of Na-MMT (25 g) was dispersed in 800 mL of distilled water, heated to 80°C with stirring for 2 hours, then centrifuged for 5 minutes to obtain the precipitate. After circulating the Na-MMT suspension for 2 hours, 10 g of (CTAB) were added. The precipitate was repeatedly washed with distilled water until no bromide ions were detected using 0.1N silver nitrate solution. Next, it was dried for 24 h at 60°C (Mohamed et al., 2020).

#### 2.1.2 Formulation of guar gum/PVA nanocomposite film

**SCHEME 1:**
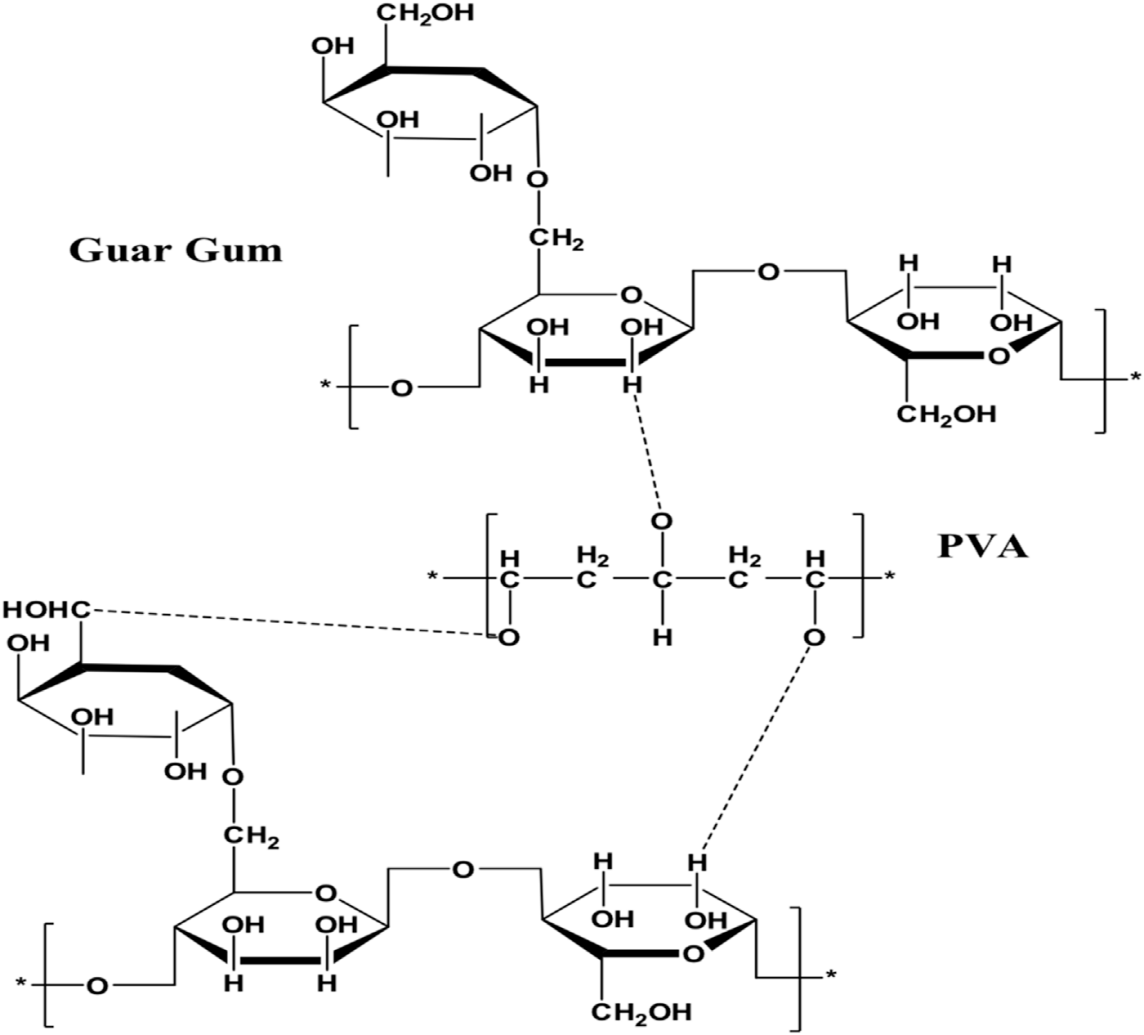
Preparation of GG/PVA/MMT nanocomposite film.

By combining several compositions that were pre-cooked, GG/PVA polymer solution combinations of diverse compositions were created using the solution casting technique (Wang and Rhim, 2015). To prevent the formation of air bubbles, 3g of guar gum (GG) was dissolved in 50°C distilled water with gentle stirring for 2 hours. In 2 hours, 2g of PVA were dissolved in distilled water at 70°C while being stirred at 1,000 rpm. After that, the guar gum solution and PVA solution were combined thoroughly and constantly stirred until total miscibility. Table 1 displays the weight percentage ratios of GG and PVA in the mixtures, which were (50/50), (60/40), and (80/20) wt%. 0.5 g of OMMT was added once the GG/PVA polymer solution was prepared. After that, 5 wt% (the total dry weight of the polymers) of glycerol was added as a plasticizer, and the mixture was vigorously stirred for 1 hour to achieve a homogenous solution. A precise volume of 20 mL of the mixture was poured into a 15 cm glass petri dish and allowed to dry in a vacuum oven for the entire night at 60°C. As indicated in Scheme 1, the films were taken out of the glass petri dish and placed in polyethylene bags to prevent contamination until needed.

**TABLE 1:** Composition of prepared GG/PVA nanocomposite film.

Sample	GG (1 wt/v%)	PVA (wt/v%)	Glycerol (wt/v%)	OMMT (g)
GG/PVAI	50	50	5	0.5
GG/PVAII	60	40	5	0.5
GG/PVAIII	80	20	5	0.5

#### 2.1.3 Formulation of metal ion solutions

After 1.0 g of copper metal (Merck, 99.99%) was solubilized in 20 mL of HNO_3_ and a few drops of HCl, a freshly produced synthetic stock solution containing 1,000 mg/L of copper ions was equipped. The solution was then diluted up to 1 L using distilled water. One liter of 7M HCl was used to dissolve 1 g of cadmium metal (Merck, 99.5%) yielding a stock solution of 1,000 mg/L cadmium. The concentration of copper and cadmium ions was approximated using Inductively Coupled Plasma-Optical Emission Spectrometer (ICP-OES, Agilent, USA).

#### 2.1.4 Instrumentation and characterization

The FTIR spectra of GG, OMMT, GG/PVAI, GG/PVAII, and GG/PVAIII were recorded using KBr discs on Perkin-Elmer Spectrophotometer to verify the formation of GG/PVA nanocomposite film. 1H NMR shifts were identified by Mestre Nova software for peaks interpretation. The prepared nanocomposite morphology and size were determined using a highly efficient technique (TEM, JEM-200CX, JEOL, Japan), which uses a high-energy electron beam to illuminate a thin sample and then interacts with it to detect the shape, size, and density of quantum wells or wires and dots. After making the sample suspension in ethanol, it was sonicated for 20 min. Next, a copper grid was loaded with a few drops of the resultant suspension at an accelerated voltage of 200 kV to investigate the sample structure. Using a TGA apparatus, samples weighing between 0.98 and 1.5 mg were packed in aluminum pots and heated to 600°C at a rate of 10°C/min in a nitrogen-filled environment. Using a controlled dry nitrogen flow rate of 20 mL/min, TGA was carried out in the temperature range of room 600°C at a rate of 20°C/min using a Shimadzu TGA-50 system from Japan.

### 2.2 Adsorption batch experiments

#### 2.2.1 Factor of pH

Cu^2+^ and Cd^2+^ ion uptake studies were carried out at regulated pH settings (1.0–7.0), with each dry nanocomposite divided into 10 mg parts and stored in separate flasks. Each flask received a 25 mL (100 mg/L) solution of either Cd^2+^ or Cu^2+^. pH was optimized using diluted HCl or NaOH solutions. The flasks were shaken on a vibromatic-384 shaker at 100 rpm for 2 hours at 25°C. Following equilibration, the metal ion residual concentration was estimated using ICP-OES and the uptake was determined using Eq. 1 (Mubark et al., 2021; Yousif et al., 2022; Mubark et al., 2023), and as follows:

$$q=\frac{\left(C_{\text{initial}}-C_{\text{final}}\right)\times \text{Volume}}{\text{Weight}}$$ (1)

q: the uptake in mg/g, C_*initial,*_ and C_*final*_ are in mg/L, Volume is in L and Weight is in g. The standard deviation was used to calculate the averaged findings after repeating each experiment three times.

#### 2.2.2 Factor of sorbent dose

The Cu^2+^ and Cd^2+^ uptake at various sorbent dosages was assessed by rotating 10–50 mg of each adsorbent nanocomposite in different flasks. Each flask contains a 25 mL (100 mg/L) Cu^2+^ or Cd^2+^ solution with a pH of 6.0. Flasks were shaken for 2 hours at 25°C. Following adsorption, the concentration of remaining metal ions in the solution was determined. The removal efficiency values were then computed using the following equation, which represents the initial and final metal ion concentrations, respectively (Eq. 2).

$$\text{Efficiency of removal}=\left[\left(C_{initial}\;–\;C_{\text{final}}\right)\;/\;C_{initial}\right]\times 100\%$$ (2)

#### 2.2.3 Factor of contact time and adsorption kinetics

Using 10 mg of each adsorbent nanocomposite in multiple flasks containing 25 mL of Cu^2+^ or Cd^2+^, at an optimal pH (pH 6.0) and a starting concentration of 100 mg/L, the uptake of Cu^2+^ and Cd^2+^ as a function of contact time (30–360 min) was measured. At 25°C and 100 rpm, the flask and its contents were shaken. For the purpose of calculating the concentration of Cu^2+^ or Cd^2+^ residuals, 5 mL of the solution were retrieved at various periods, filtered and measures using ICP-OES. Uptake was calculated via Eq. 1 as previously described.

The stirring time parameter data were used to investigate the adsorption kinetics of the three produced GG nanocomposites for the metal ions under study. This was carried out by applying the results using kinetic models equations, namely,; pseudo-1^st^-order, pseudo-2^nd^-order.

The two models were represented as follows in Eqs. (3, 4) (Weber and Morris, 1963; Ho and McKay, 2019; Eliwa and Mubark, 2021; Mubark et al., 2022a)

$$\log\left(q_{e}-q_{t}\right)=\log{\;q}_{e}-\left(\frac{k_{1}}{2.303}\right)t$$ (3)

$$\frac{t}{q_{t}}=\frac{1}{k_{2}q_{e}^{2}}+\frac{1}{q_{e}}t$$ (4)

k_1_ and k_2_: the reaction rate constants for the pseudo-1^st^-order, pseudo-2^nd^-order, respectively. q_e_ and q_t_:the amounts of Cu^2+^ and Cd^2+^ adsorbed at equilibrium and at time t, respectively.

#### 2.2.4 Factor of temperature and adsorption isotherms

Cu^2+^ and Cd^2+^ adsorption isotherms were derived by adding 10 mg of each adsorbent nanocomposite to a series of flasks holding 25 mL of the respective adsorbent at a specific concentration (20, 30, 40, 50, 70, and 100 mg/L) and pH 6.0 for 240 min. Temperatures were maintained at 25, 35, 45, and 55°C while the flasks were shaken at 100 rpm. The residual Cu^2+^ and Cd^2+^ concentration was measured following equilibration.

Two adsorption isotherms were utilized to explain the adsorption mechanism of both metal ions on the prepared nanocomposites, namely, Langmuir and Freundlich models. The empirical model’s equations were represented as follows in Eqs. (5, 6)

$$\frac{C_{e}}{{\;q}_{e}}=\frac{C_{e}}{Q_{\max}}+\frac{1}{Q_{\max}{\;K}_{L}}$$ (5)

$$\log{\;q}_{e}=\log K_{f}+\frac{1}{n}\;\log{\;C}_{e}$$ (6)

Q_max_, k_L_, and C_e_: the maximum adsorption capacity, the Langmuir constant is related to the adsorption capacity, and the metal ion concentration in solution. K_f_ and 1/n: the Freundlich constant related to adsorption capacity and intensity, respectively.

#### 2.2.5 Factor of desorption and reusability studies

EDTA solutions were used to set up the desorption experiments. To remove any unabsorbed species, 10 mg of the loaded adsorbent nanocomposite containing Cu^2+^ or Cd^2+^ ions was carefully washed with distilled water. After that, the nanocomposite was shaken for 3 hours at 100 rpm and 25 °C using 25 mL of 1% EDTA. Following a mild washing with distilled water, the nanocomposite was examined to see if it could be used again in the subsequent uptake run. The following equation (Eq. 7) served as the basis for determining the tendency efficiency for the re-uptake.

$$\text{Regeneration efficiency }\left(\%\right)=\frac{\text{uptake}\;\text{in}\;\text{the}\;2^{\text{nd}}\;\text{run}}{\text{uptake}\;\text{in}\;\text{the}\;1^{\text{st}}\;\text{run}}\times 100\;\%$$ (7)

#### 2.2.6 Application of the studied nanocomposites for a real sample

To investigate the applicability of the prepared nanocomposites to remove Cu^2+^ and Cd^2+^, 100 mL portions of wastewater were obtained from Nuclear Material Authority (NMA) laboratories from different laboratories. Using prepared nanocomposites, this wastewater was processed by implementing the most beneficial controlling factors influencing the adsorption of Cu^2+^ and Cd^2+^ at room temperature. According to the methodology described above, estimation of concentration of Cu^2+^ and Cd^2+^ was performed before and after treatment.

## 3 Results and discussion

The nanocomposite film was synthesized by casting solution method through blending of GG and PVA as mentioned in Table 1. Together with the carboxylic acid groups of GG, the hydroxyl groups of PVA can form hydrogen bonds (Scheme 1). Hydrogen bonds are dynamic bonds that have the potential to provide desirable qualities like toughness, self-healing, and shape memory (You et al., 2020). While other aggregates, such as micelles and inorganic fillers, have varying sizes at the nano or micron level and obstruct optical properties, hydrogen bonds are stable in the presence of ions. Hydrogen bond-based films have the potential to be transparent, making them ideal for use as packaging material in sensors and electronics. H-bonds, however, are unable to obtain appropriate overall mechanical properties. They can be robust and brittle or soft and tough. Thus, we present a novel nanocomposite GG/PVA film that was created by blending PVA and GG in a cast solution while employing glycerol as a plasticizer. Plasticizers were utilized to enhance the film’s characteristics. The molecule of plasticizer enters among the molecules of GG and PVA causing the reduction of the intermolecular force of attraction in addition to its role in the formation of hydrogen bonds with GG and PVA. The crosslinking agents can form ether linkages through the reaction with the available hydroxyl groups found in GG and PVA, which assist in the acceleration of the film’s mechanical properties.

### 3.1 Characterization of prepared nanocomposite films

#### 3.1.1 FT-IR results

To identify the chemical bonds within a molecule, this analysis is dependent on the generation of an infrared absorption spectrum (Chen et al., 2015; Mubark et al., 2022b). The FT-IR provides a full and detailed analysis of the sample through screening and scanning it for many various components to determine its characteristic molecular fingerprint. It is also an efficient way to determine the functional groups and the covalent bond (Rohman et al., 2020).

**FIGURE 1:**
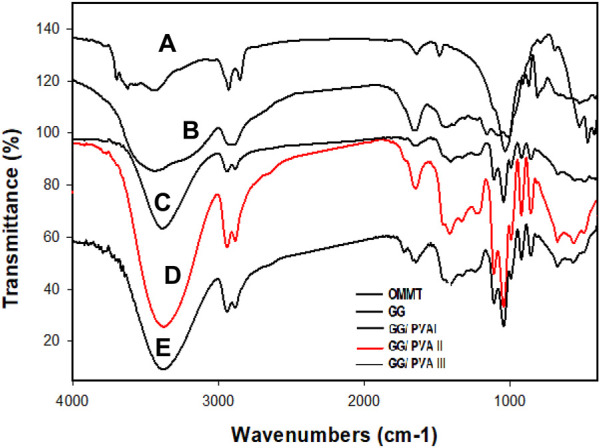
FT-IR analysis of **(A)** OMMT, **(B)** GG, **(C)** GG/PVAI, **(D)** GG/PVAII, and **(E)** GG/PVAIII.

The FTIR analysis of OMMT, GG, GG/PVAI, GG/PVAII, and GG/PVAIII was obtained in Figure 1. Bands at 2,922, 2,851, and 1,469 cm^-1^ in OMMT’s Figure 1A were attributed to the stretching vibration of N-H, the symmetric stretching vibration of the C-H bond of -CH_2_, and the sharp peak of C-H bending vibration absorption of CTAB, separately which verified the modification of MMT (Verma et al., 2013). The FTIR for the native GG is displayed in Figure 1B. A wide band at 3,444 cm^-1^ was visible on the chart, which corresponded to -OH stretching vibrations and H_2_O involved in the formation of hydrogen bonds. A tiny band was identified as the -CH stretching vibration at 2,925 cm^-1^. The bands recognized in the spectrum between 800 and 1,200 cm^−1^ represented the highly coupled C-C-O, C-OH, and C-O-C stretching modes of the backbone of the polymer. The FT-IR spectra of GG/PVA films are shown in Figures 1C–E. The chosen films that were subjected to FT-IR analysis revealed commonalities and overlaps. Furthermore, it was shown that the stretching vibration areas of the hydrogen group are connected to the strong broad band that appeared between 3,700 and 3,000 cm^-1^ (Fan et al., 2012). The -OH band can undergo an FT-IR wavenumber transformation because it is extremely sensitive to hydrogen bonding. When GG/PVA film spectra are compared at 3,400 cm^-1^, the sharpened and expatriate bands appeared at a higher wavenumber, indicating a shift in the glycerol and PVA content. The formation of various types of hydrogen bonds, both intra- and intermolecular, is linked to the presence of -OH groups in prepared films. The shift was therefore observed at 3,400 cm^-1^ as a result of the hydrogen bonding interactions between the GG-glycerol-PVA molecules (Acharya et al., 2017). Vibration bands at 2,902 and 2,938 cm^-1^ are caused by aliphatic C-H stretching from alkyl groups, and they intensify in the presence of PVA and glycerol. The vibration bands at 2,902 and 2,938 cm^-1^ are mostly caused by the aliphatic C-H stretching from alkyl groups, which becomes more intense when glycerol and PVA are present.

#### 3.1.2 NMR spectroscopy

**FIGURE 2:**
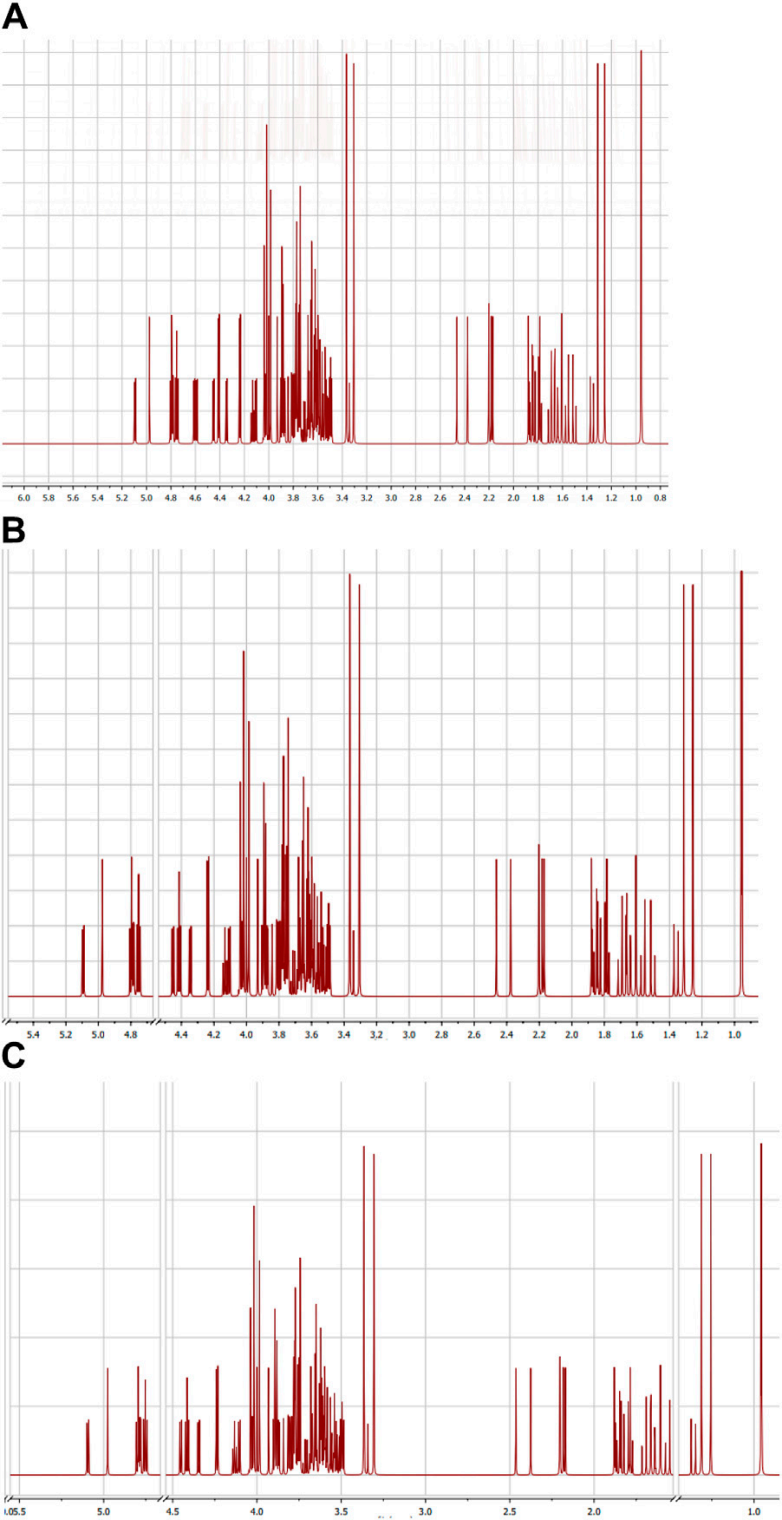
NMR analysis of **(A)** GG/PVAI, **(B)** GG/PVAII, and **(C)** GG/PVAIII.

1 H-NMR spectroscopy, which is presented in Figure 2, was also used to confirm the structures of GG/PVAI, GG/PVAII, and GG/PVAIII. A peak with δ = 1.28–1.33 ppm was observed in 1H-NMR analysis for CH2−methylene protons attached to the–O– group, and 1.88–2.2 ppm were observed for protons of (–C–O–H) alcohol substituted to GG at side wise coupled to the cyclic ring carbon. The peaks that developed between 3.50 and 3.78 ppm were caused by methylene protons that were bound to carbons in the formation of cyclic rings and had alcohol on the other side of those protons. The -CH2 protons (d), which are attached to the cyclic carbon ring attached to the side and terminal side -O-H functional group employed in grafting, are responsible for the peaks that appeared in the range of 3.88–4.15.

#### 3.1.3 TEM results

**FIGURE 3:**
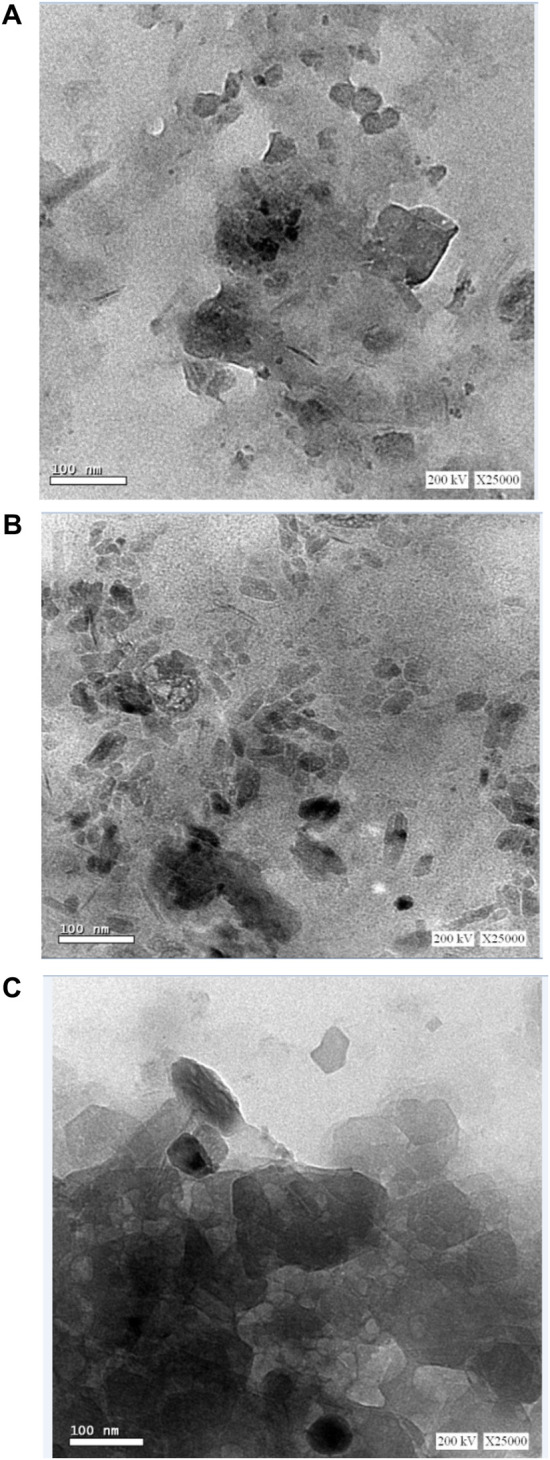
TEM of GG/PVA **(A)** 50/50) **(B)** (60/40), **(C)** (80/20)

The characteristics of extremely small specimens are observed using TEM analysis. It is known that there are various types of nanometer-sized particles; therefore, the utilization of TEM permits obtaining information concerning the particle size, shape, and surface layers. So atomic structures can be directly imaged in solids and surfaces by TEM. It is utilized in the first place to determine the presence of prepared metal particles on a nano-scale and it is also very useful in estimating the size of the metal formed nanoparticles (Brodusch et al., 2021). The distribution of OMMT nano clay particles into GG/PVA film could be clarified by using transmission electron microscopy (TEM), shown in Figure 3. The small spherical point groups were localized by TEM, which appears to be superbly dispersed in the GG/PVA films. The image also represents some amount of collections in some regions, which may be due to the presence of a GG/PVA polymer blend responsible for the stabilization of nano clay particles as suggested by the small spherical point groups localized by TEM, and the GG/PVA films seem to have excellent dispersion. The image also shows some collections in some areas, which could be explained by the existence of a GG/PVA polymer mixture that stabilizes nano clay particles (Awadeen et al., 2020).

#### 3.1.4 TGA analysis

**FIGURE 4:**
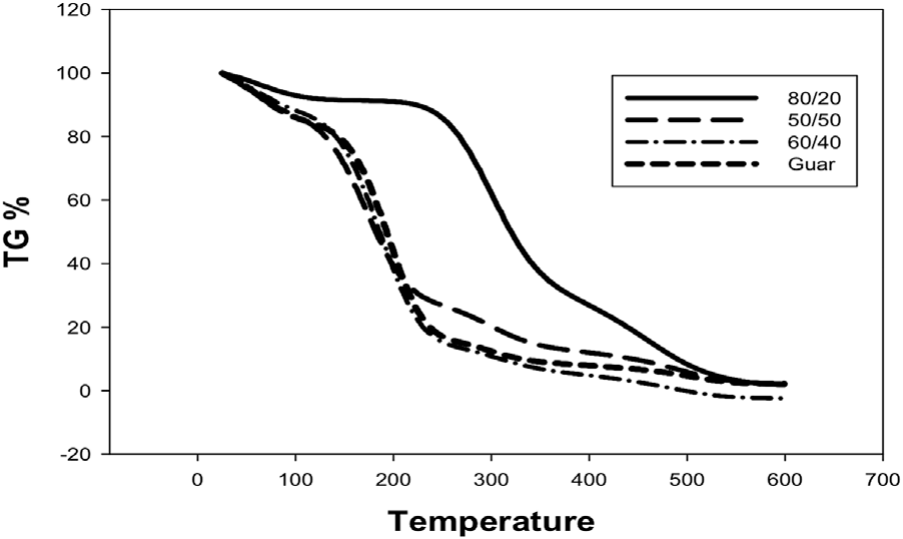
TGA of Prepared guar and GG/PVA 50/50), (60/40), and (80/20)

The effects of adding nano clay surface-modified montmorillonite to guar gum on its thermal stability were investigated using TGA analysis. The weight loss (%) over a broad temperature range of 100–600°C was used to assess the thermal stability of guar gum and montmorillonite, as illustrated in Figure 4. It was recognized that the decomposition rate was lower in the case of nanocomposite than native guar. The chemical modification of guar was detected by the presence of a three-stage decomposition mechanism and by the observation of maximal weight loss after the decomposition reaction of the first stage in the nano samples. The initial decomposition temperature (IDT), which refers to the point at which moisture or volatile compounds were lost, was between 100°C and 200°C in each case. Because of the chain elimination, the secondary stage was identified in the 300–500°C temperature range. Ultimately, the fracture of the composites’ structure at 600°C led to the determination of the third stage. In regarding thermal stability, guar gum (80/20) exceeded the other composites tested. This is owing to the existence of a higher percentage of guar gum and a lesser percentage of clay, as the latter’s high ratio has a negative impact on a specific proportion due to its collapse at high temperatures. This could be connected to the presence of guar gum at a higher ratio than MMT, which has a deleterious effect on the manufactured composites. High-temperature thermal treatment of clay minerals frequently results in the formation of new microcracks or the extension/widening of pre-existing microcracks, resulting in variations in physical attributes (Sun et al., 2016). Upon comparing with the native guar, the decomposition rate was lower in the case of composites than the native guar, which indicates the more compact and maintained polymeric network in nanocomposites. This could be a result of the polymeric network’s incorporation of nano clay, which acts as a barrier to prevent both mass and energy conveyance in the composite network.

### 3.2 Measurements of uptake carried out via batch method

#### 3.2.1 Results of pH factor

**FIGURE 5:**
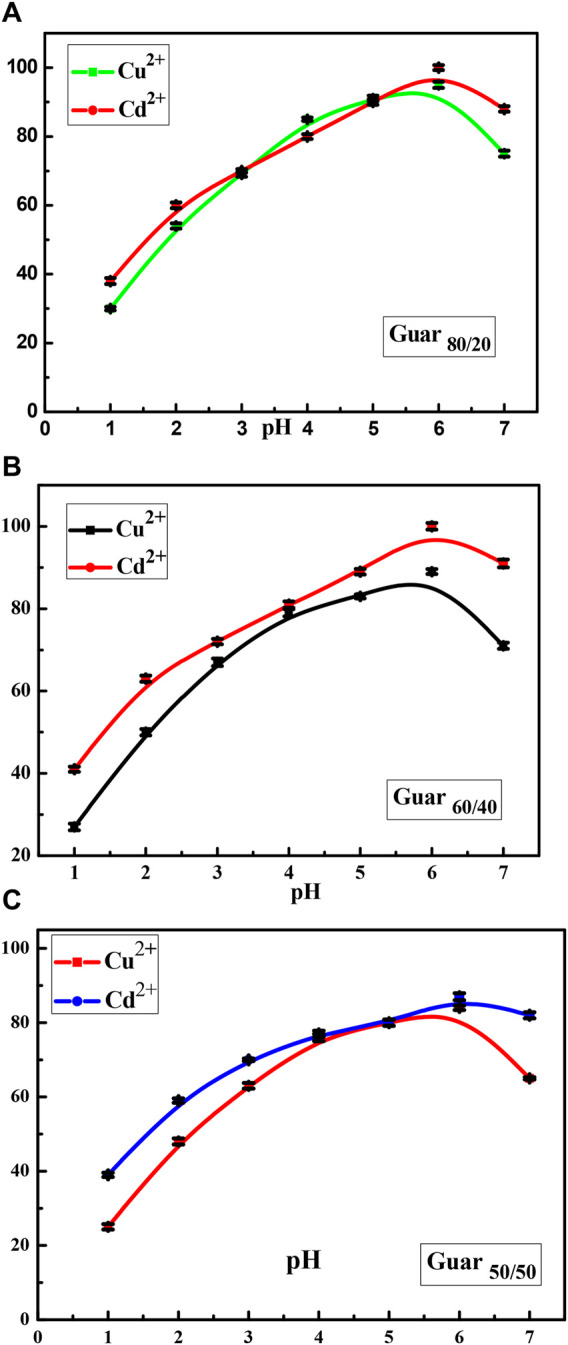
pH factor of Cu^2+^ and Cd^2+^ ions adsorption on different prepared GG/PVA **(A)** (80/20), **(B)** (60/40), and **(C)** (50/50)

Figure 5 shows the extent to which the three prepared nanocomposites were able to adsorb Cu^2+^ or Cd^2+^ ions from liquid solutions depending on the pH effect in non-competitive conditions. In every instance, the maximum uptake for those metal ions was found at pH 6.0. When the effectiveness of the variously prepared nanocomposites was compared, the modified nanocomposite with an 80/20 ratio demonstrated the greatest removal across the entire pH range. According to the majority of research, the pH range of 4.0–6.0 is where Cu^2+^ and Cd^2+^ adsorption results in the highest removal percentage (Fu and Wang, 2011; Carolin et al., 2017). Because protons and metal ions compete for adsorption sites, the extraction efficiency for both metal ions with the various nanocomposites was lowest at low pH. As the pH increased, the solution’s pronation decreased and uptake increased accordingly. The hydrolysis of both metal ions caused a decrease in the extraction percentages of Cu^2+^ and Cd^2+^ at pH values greater than 7.0 (Lü et al., 2013). Thus, in the following studies, pH 6.0 was selected for the removal of Cu^2+^ and Cd^2+^. The mechanism of the interaction of these metal ions with the prepared nanocomposites were expected to achieve through the interaction of the free hydroxyl groups present on the surface of the nanocomposites.

#### 3.2.2 Results of sorbent dose factor

**FIGURE 6:**
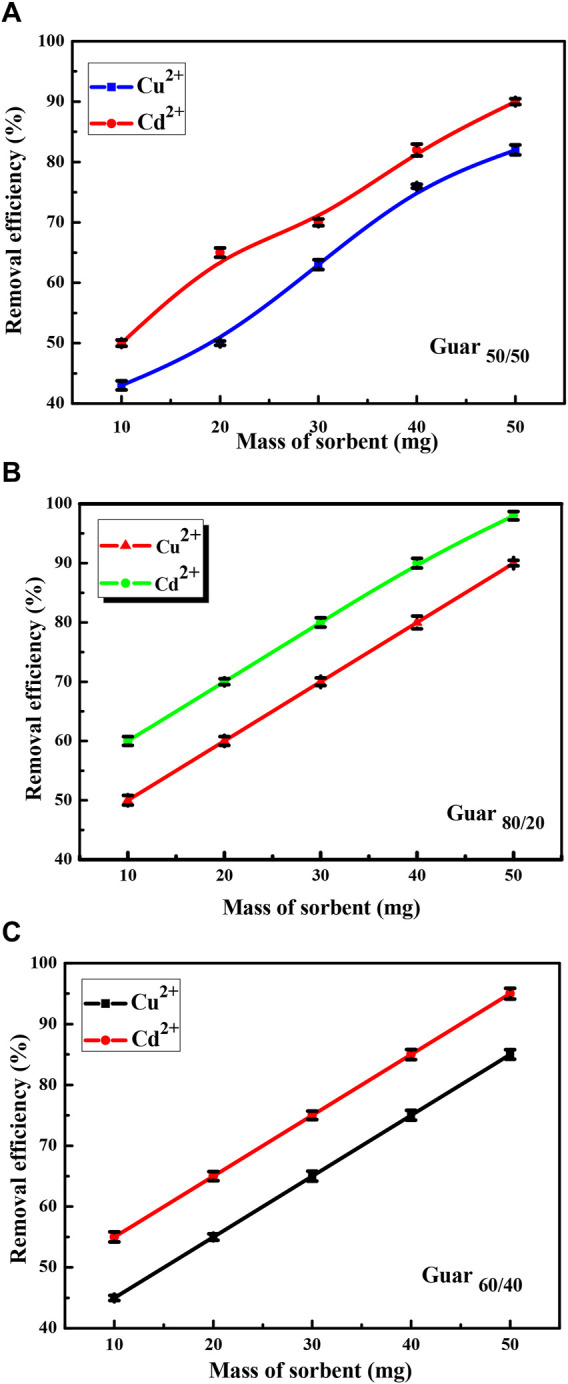
Sorbent dose factor of Cu^2+^ and Cd^2+^ adsorption on different Prepared GG/PVA **(A)** 50/50), **(B)** (80/20), and **(C)** (60/40)

At pH 6.0, the effects of sorbent dose (sorbent dose 10–50 mg, initial metal ion concentration 100 mg/L) on the potency of Cu^2+^ and Cd^2+^ ion elimination onto the three distinct nanocomposites were explored. The data displayed in Figure 6 illustrates how increasing the sorbent dose had a positive impact on removal efficiency which was explained in terms of the quantity of more active adsorption sites for Cu^2+^ and Cd^2+^ in the nanocomposites.

#### 3.2.3 Results of contact time factor

**FIGURE 7:**
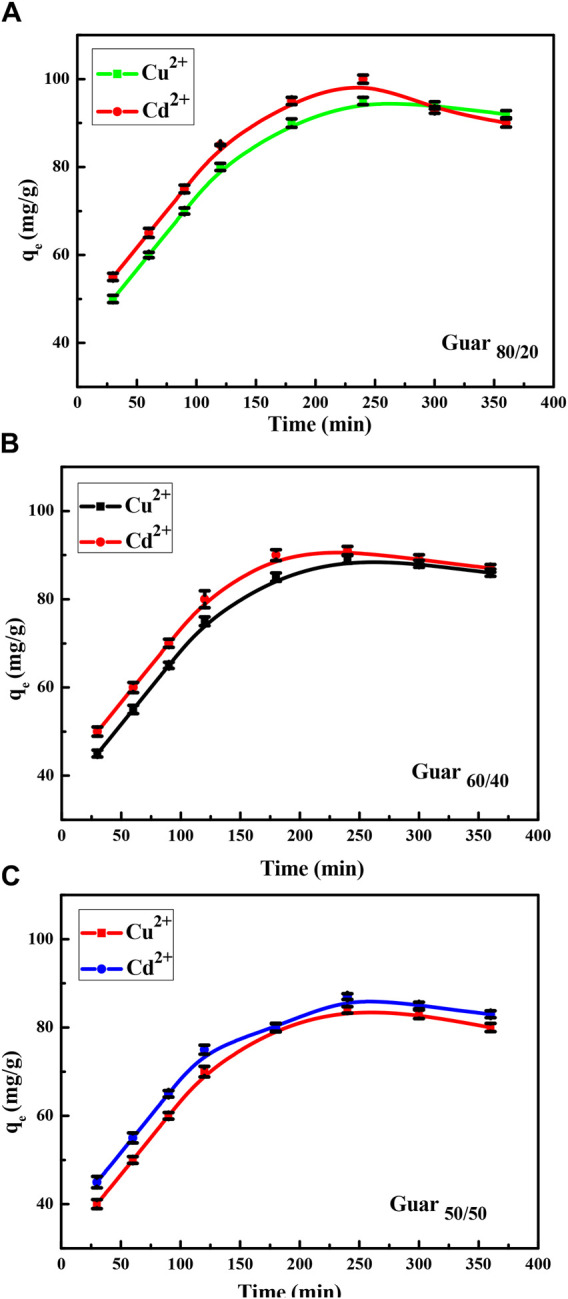
Time factor of Cu^2+^ and Cd^2+^ adsorption on different Prepared GG/PVA **(A)** 80/20), **(B)** (60/40), and **(C)** (50/50).

Figure 7 shows the uptake measurement over a fixed time period. Over the course of 120 min, the uptake of Cd^2+^ and Cu^2+^ demonstrated high uptake capacities of roughly 60% at the plateau. Within 240 min, the equilibrium uptake capacities were reached.

**FIGURE 8:**
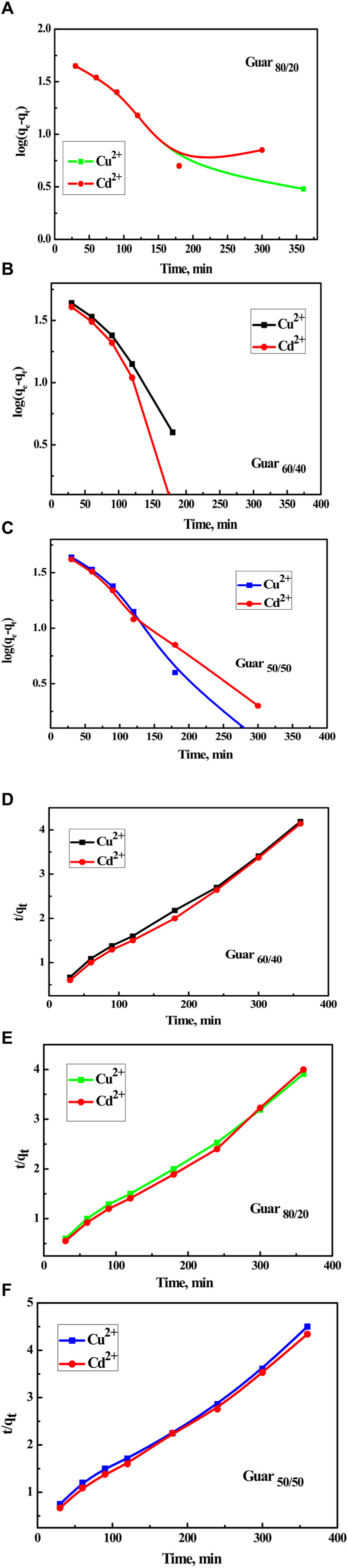
Kinetics for Cu^2+^ and Cd^2+^ adsorption on different Prepared GG/PVA Time with log(q_e_-q_t_) **(A) **80/20), Cu^2+^ **(B)** (60/40), and Cd^2^ **(C)** (50/50). Time with (t/q_t_) **(D) **60/40), **(E)** (80/20), and **(F)** (50/50).

To examine kinetics of the adsorption reaction, pseudo-1^st^-order and pseudo-2^nd^-order models were applied to the adsorption data. The pseudo-1^st^-order could model the adsorption process as one component dependent, albeit the pseudo-1st-order model was being properly managed for the bimolecular process. In short, adsorbent or adsorbate amounts mastered the pseudo-1^st^-order-model. In many cases, the pseudo-1^st^-order-model mainly describes adsorption as a physical process entirely physisorption. The kinetic parameters were produced from the linear plot of log q_e_ vs. log(q_e_-q_t_) for pseudo-1^st^-order and (t/q_t_) vs. t for pseudo-2^nd^-order as shown in Figure 8, respectively. Fit of the straight line (R^2^) and consistency between calculated and experimental values of q_e_, as shown in Tables 2, 3 for Cu^2+^ and Cd^2+^ ions, respectively, were used to assess each sample’s efficacy. As a result, the pseudo-2^nd^-order model’s estimation of the theoretical adsorption uptakes of both metal ions on various nanocomposites was more accurate than the pseudo-1^st^-order model’s calculation. pseudo-2^nd^-order shows that there is a chemical process involved in the adsorption between the adsorbent and the adsorbate, meaning that the ions Cu^2+^ and Cd^2+^ adsorb on the synthesized nanocomposites chemically.

**TABLE 2:** Kinetics parameters of Cu^2+^ adsorption on different nanocomposite films.

Sorbent	Pseudo-1st-order					Pseudo-2nd-order		
	q_e_ (exp) (mg/g)	K_1_ (min^–1^)	q_e_ (calc) (mg/g)	Error bar	R^2^	K_2_ (g/mg.min)	q_e_ (calc) (mg/g)	R^2^
Guar (50/50)	84	10^−2^ × 1.6	86.02	0.6549	0.957	10^−4^ × 2.59	93.11	0.990
Guar (60/40)	89	10^−2^ × 1.6	86.02	0.8165	0.957	10^−5^ × 2.61	98.23	0.994
Guar (80/20)	95	10^−3^ × 8.544	47.58	0.5	0.849	10^−4^ × 2.60	104.6	0.994

**TABLE 3:** Kinetics parameters of Cd^2+^ ions adsorption on different nanocomposite films.

	Pseudo-1st-order					Pseudo-2nd-order		
Sorbent	q_e_ (exp) (mg/g)	K_1_ (min^–1^)	q_e_ (calc) (mg/g)	Error bar	R^2^	K_2_ (g/mg.min)	q_e_ (calc) (mg/g)	R^2^
Guar (50/50)	87	10^−2^ × 1.14	86.02	0.6236	0.988	10^−4^ × 3.14	93.72	0.994
Guar (60/40)	91	10^−2^ × 2.4	71.21	0.7348	0.887	10^−3^ × 3.68	96.99	0. 992
Guar (80/20)	100	10^−3^ × 7.738	45.32	0.7364	0.776	10^−4^ × 2.800	100.6	0.986

#### 3.2.4 Results of temperature and adsorption isotherms factor

**FIGURE 9:**
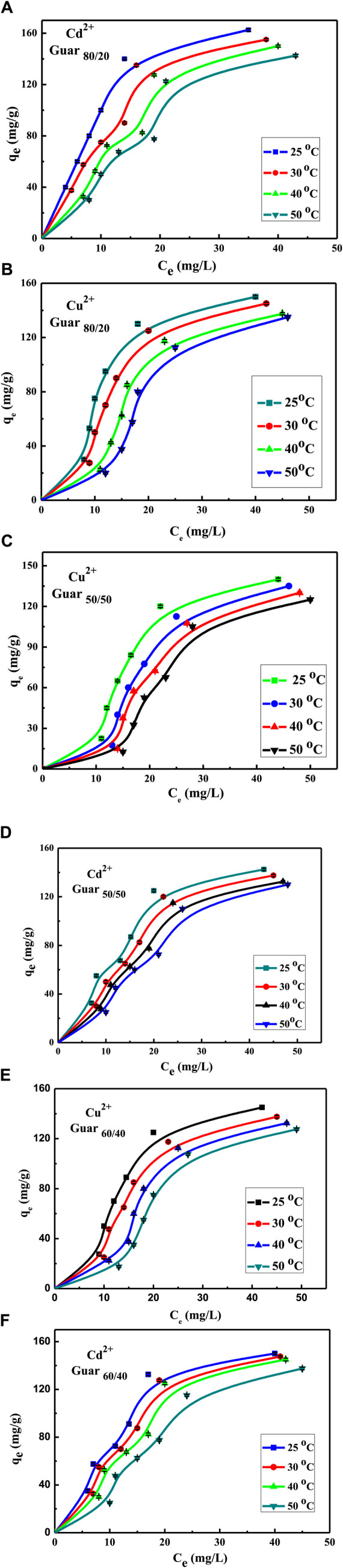
Concentration factor of Cu^2+^ and Cd^2+^ adsorption on different GG/PVA Cd^2^ **(A)** 80/20), Cu^2+^ **(B)** (80/20), and Cu^2^ **(C)** (50/50). **(D) **50/50) Cd^2^, Cu^2^ **(E)** (60/40), and Cd^2^ **(F)** (60/40).

Figure 9 shows both metal ions adsorption isotherms on the assessed guar gum nanocomposites at different temperatures. At 25°C, it emerged that the maximum uptake values of the various films that were prepared for Cu^2+^ were 95, 89, and 84 mg/g for guar gum (80/20, 60/40, and 50/50), respectively. However, at 25°C, the maximum uptake values for Cd^2+^ in guar gum (80/20, 60/40, and 50/50), respectively, were found to be 100, 91, and 87 mg/g. Uptake rose until equilibrium was reached at a known temperature as the equilibrium concentration increased.

**FIGURE 10:**
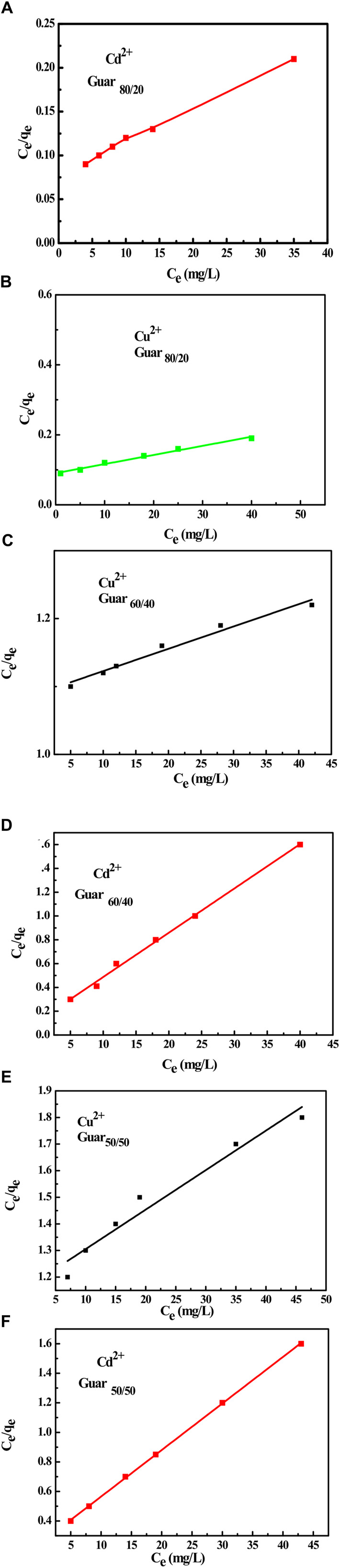
Langmuir isotherm for Cu^2+^ and Cd^2+^ adsorption on different GG/PVA Cd^2^ **(A) **80/20), Cu^2+^ **(B)** (80/20), and Cu^2+^ **(C)** (60/40). Cd^2^ **(D) **60/40) Cu^2^, **(E)** (50/50), and Cd^2^ **(F)** (50/50).

The equilibrium adsorption isotherms are the key to understanding the metal ion adsorption mechanism on the prepared guar gum film. The Langmuir and Freundlich isotherms are typically used to characterize the adsorption process (Savaskan Yilmaz et al., 2020). To estimate the removal efficiency for different sets of initial solute concentrations, solution volumes, and adsorbent masses or to forecast the adsorbent mass required for solute removal at in-demand retrieval efficiency—the intrinsic parameter of the Langmuir and Freundlich adsorption isotherms was obtained (Chung et al., 2015; Dakroury et al., 2022). Since the Langmuir equation is believed to be the most widely used isotherm equation for modelling equilibrium data, it was applied to the collected adsorption data. As shown in Figure 10, a straight line with slope and intercept equal to 1/Q_max_ and 1/K_L_Q_max_, respectively, was obtained by plotting C_e_/q_e_ against C_e_. Measurements were made of the slopes and intercepts for both metal ions adsorption, and the resulting values of Q_max_ and K_L_ are reported in Tables 4, 5, respectively, for Cu^2+^ and Cd^2+^. The Q_max_ values are compared to the experimental values, and the Langmuir model was found to provide a better fit for the experimental data based on the correlation coefficient R^2^ values. These Langmuir model-dependent results demonstrated that monolayer coverage essentially determines the adsorption process, which was validated (Russakova et al., 2021).

**TABLE 4:** Parameters of Cu^2+^ adsorption isotherm on different nanocomposite films.

Sorbent	Langmuir			Freundlich		
	Q_max_ (mg/g)	K_L_ (L/mg)	R^2^	K_f_ (mg/g)	n	R^2^
Guar (50/50)	67.5	0.013	0.953	2.56	0.88	0.701
Guar (60/40)	304	0.03	0.972	1.81	0.84	0.737
Guar (80/20)	384	0.03	0.986	1.09	0.75	0.721

**TABLE 5:** Parameters of Cd^2+^ adsorption isotherm on different nanocomposite films.

Sorbent	Langmuir			Freundlich		
	Q_max_ (mg/g)	K_L_ (L/mg)	R^2^	K_f_ (mg/g)	n	R^2^
Guar (50/50)	31.72	0.13	0.999	6.38	1.15	0.850
Guar (60/40)	26.86	0.324	0.996	9.03	1.23	0.823
Guar (80/20)	263.85	0.05	0.996	1.09	0.75	0.731

**FIGURE 11:**
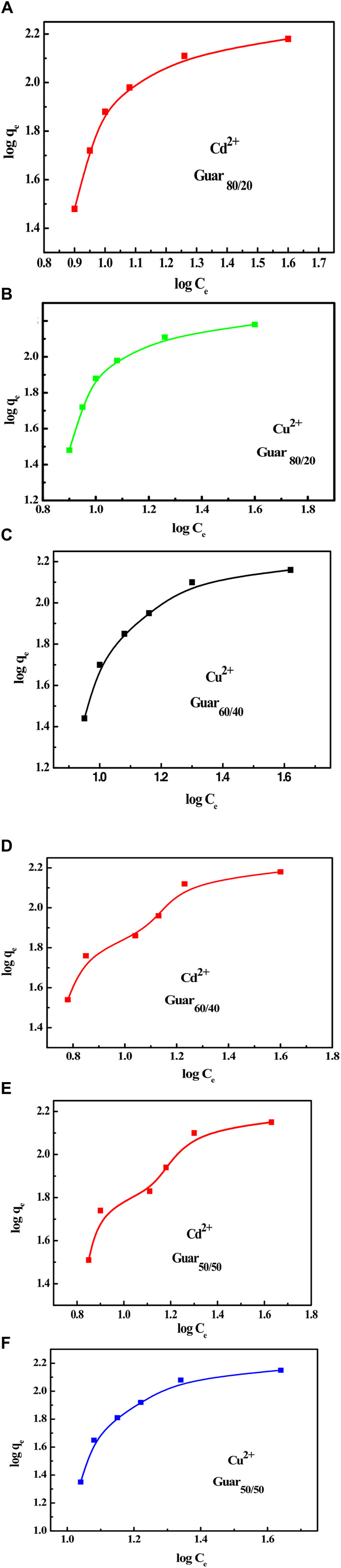
Freundlich isotherm for Cu^2+^ and Cd^2+^ adsorption on different Prepared GG/PVA Cd^2^ **(A) **80/20), Cu^2+^ **(B)** (80/20), and Cd^2^ **(C)** (60/40). Cu^2^ **(D)** 60/40) Cd^2^, **(E)** (50/50), and Cu^2^ **(F)** (50/50).

From the adsorption data for both metal ions, the Freundlich isotherm was computed by graphing log q_e_ vs log C_e_, which results in a straight line with slope and intercept values equal to 1/n and log K_f_, respectively, as shown in Figure 11. The obtained values of K_f_, n, and R^2^ for both metal ions with different guar gum nanocomposites were displayed in Tables 4, 5 for Cu^2+^ and Cd^2+^, respectively. These results suggest that the adsorption process is consistent with the Langmuir model. These findings proved that both metal ion adsorption on the different guar gum nanocomposites was accomplished as a monolayer on the homogenous sites of the guar gum nanocomposites and that this process was also demonstrable (Huang and Keller, 2020; Ahmed et al., 2021).

#### 3.2.5 Regeneration of nanocomposites

As the revival of the nanocomposites loaded by Cu^2+^ or Cd^2+^ ions was processed using 1% EDTA, regeneration of the loaded nanocomposites by Cu^2+^ or Cd^2+^ was explored (Huang and Keller, 2020). Because of its numerous functional groups, EDTA has a high capacity to form a complex with these two metal ions. The mechanism of the reaction between metal ions and EDTA was shown by Eq. 8. With a standard deviation of ±2%, the regeneration of nanocomposite efficiency was reported to be 95% across two cycles, suggesting the stability of the developed nanocomposites even after repeated use.

$$M_{\left(\text{aq}\right)}^{2+}+{\left(\text{EDTA}\right)}_{\left(\text{aq}\right)}^{2–}\to {M{\left(\text{EDTA}\right)}^{2–}}_{\left(\text{aq}\right)}+{2H^{+}}_{\left(\text{aq}\right)}$$ (8)

Where $M^{2+}={\text{Cu}}^{2+}$ or ${\text{Cd}}^{2+}$

#### 3.2.6 Application of prepared guar gum nanocomposites towards wastewater treatment

Inquiring into whether the produced guar gum nanocomposite films can be used to retrieve Cu^2+^ and Cd^2+^ ions from actual samples. The various nanocomposite films were used on wastewater that was obtained from NMA’s laboratories. The wastewater was treated individually with each prepared nanocomposite, based on the application of optimal controlling factors that influence the adsorption of both metal ions (pH 6.0, 4h, 25°C). The retrieval of both metal ions from the wastewater before and after the adsorption process was found to be 95.1%, 93.3%, and 91.3% for guar gum (80/20, 60/40, and 50/50), respectively, according to chemical analysis of the wastewater. Based on the previously obtained and discussed data, it was evident that the guar gum nanocomposites (Figure 11) were effective in removing copper and cadmium from wastewater with an effectiveness level above-average 90% due to their chemically stable properties and simplicity of synthesis. A study included comparison between the prepared guar gum nanocomposites with various adsorbents is reported in Table 6 (Suresh et al., 2014; Ahmad and Haseeb, 2015; Ahmad and Hasan, 2016; Ahmad et al., 2017; Yang et al., 2019; Mubark et al., 2022b).

**TABLE 6:** Comparative study of adsorption capacity for Cu(II) and Cd(II) ions with various adsorbents.

Element	Adsorbent	Uptake (mg/g)	Ref.
Cd(II)	GGH	99	Mubark et al. (2022b)
	Wood Apple Shell	32.7	Yang et al. (2019)
	L-cystein modified bentonite-cellulose nanocomposite	16.12	Ahmad and Hasan (2016)
	chitosan grafted polyaniline-OMMT nanocomposite	54.64	Ahmad et al. (2017)
	guar gum (80/20, 60/40 and 50/50)	100, 91,87	Present work
Cu(II)	Functional chitosan gel material (FCG)	75.40	Suresh et al. (2014)
	L-cystein modified bentonite-cellulose nanocomposite	32.36	Yang et al. (2019)
	Luffa acutangula modified Tetraethoxysilane (LAP-TS)	12	Ahmad and Haseeb (2015)
	GGH	90.3	Mubark et al. (2022b)
	guar gum (80/20, 60/40 and 50/50)	95, 89,84	Present work

## 4 Conclusion

Three guar gum nanocomposites were prepared and investigated towards the removal of Cu^2+^ and Cd^2+^ ions from solutions. The adsorption capacities for Cu^2+^ were found to be 95, 89 and 84 mg/g for guar gum (80/20, 60/40 and 50/50), respectively. While for Cd^2+^ was found to be 100, 91, 87 mg/g for guar gum (80/20, 60/40 and 50/50), respectively at 25°C. The interest in using of these synthesized nanocomposite for the elimination of Cu^2+^ and Cd^2+^ ions from wastewater solutions of the petroleum industries was related to its unique characteristic. The results revealed that the adsorption reaction followed PSO model. In addition to the achievement of the equilibrium of adsorption within 4 h. Moreover, the nanocomposite loaded by metal ions could be reused for repeated times by using 1% EDTA.

## Data Availability

The original contributions presented in the study are included in the article/Supplementary material, further inquiries can be directed to the corresponding authors.
